# 7-Benzyl-3-methyl-6-phenyl­imidazo[2,1-*b*][1,3]thia­zol-7-ium chloride 0.75-hydrate

**DOI:** 10.1107/S1600536813018795

**Published:** 2013-07-13

**Authors:** Huang Guo-Li, Liu Bo, Kou Jun-Feng

**Affiliations:** a School of Chemistry and Chemical Engineering, Yunnan Normal University, Knuming 650050, People’s Republic of China

## Abstract

Theasymmetric unit of the title salt, C_19_H_17_N_2_S^+^·Cl^−^·0.75H_2_O, contains two symmetrically independent formula units of the carbenium salt along with three water mol­ecules. The water mol­ecules are only 50% occupated, and one of them is positioned in a hydro­phobic pocket not forming any hydrogen bonds. The conformation of the independent cations is very similar, with dihedral angles of 61.0 (2) and 61.5 (3)° between the benzene rings. They form quasi-centrosymmetric couples *via* π–π stacking inter­actions between the benzene and imidazo[2,1-*b*]thia­zole rings [centroid–centroid distances = 3.718 (3) and 3.663 (3) Å]. In the crystal, O—H⋯Cl hydrogen bonds lead to the formation of a helical anion–water chain along the *c-*axis direction. The cations connect to the anion–water chain through C—H⋯Cl inter­actions, generating a three-dimensional supra­molecular network. O—H⋯S hydrogen bonds and C—H⋯O inter­actions also occur.

## Related literature

For applications in catalysis of abnormal *N*-heterocyclic carbenes, see: Mattson *et al.* (2006 ▶); Liu *et al.* (2008 ▶); Padmanaban *et al.* (2011 ▶). For related structures, see: Huang *et al.* (2011 ▶); Akkurt *et al.* (2011 ▶, 2007 ▶); Song *et al.* (2008 ▶).
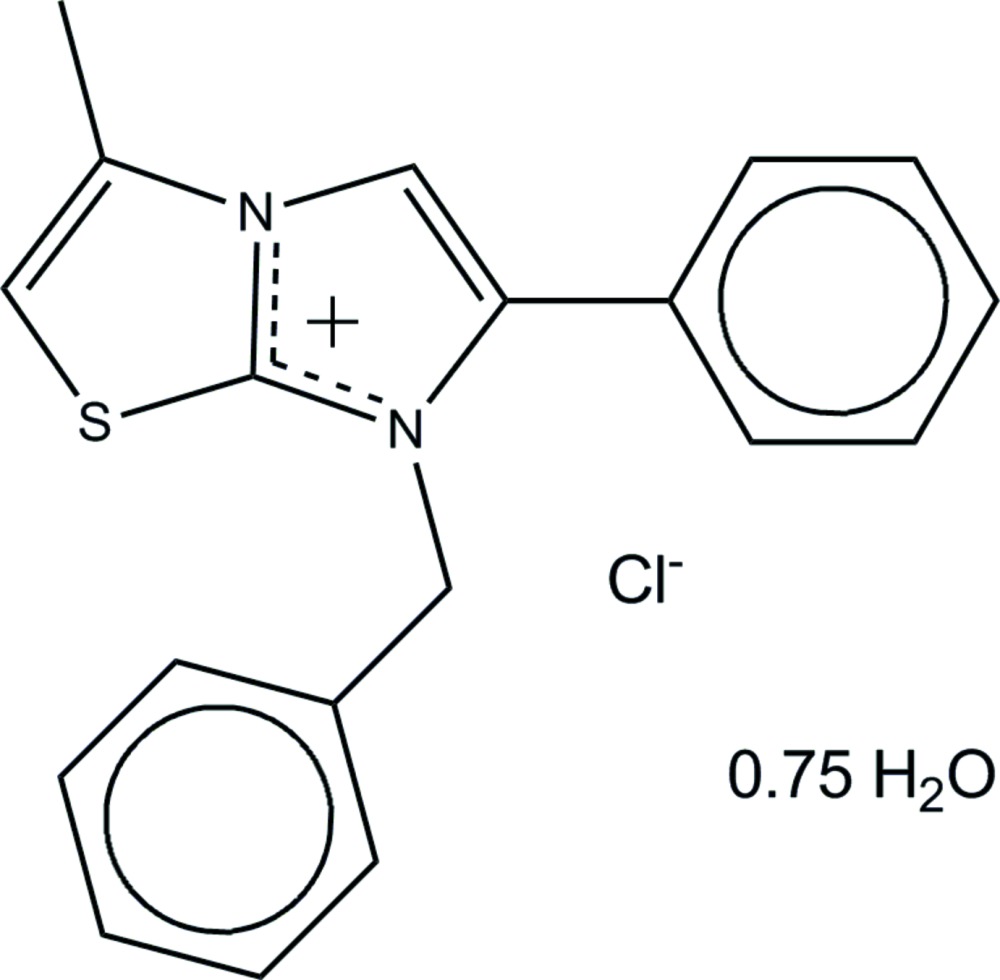


## Experimental

### 

#### Crystal data

C_19_H_17_N_2_S^+^·Cl^−^·0.75H_2_O
*M* *_r_* = 354.37
Trigonal, 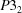

*a* = 13.211 (1) Å
*c* = 19.555 (3) Å
*V* = 2955.7 (6) Å^3^
*Z* = 6
Mo *K*α radiation
μ = 0.31 mm^−1^
*T* = 291 K
0.28 × 0.24 × 0.22 mm

#### Data collection

Bruker SMART APEX CCD diffractometer
Absorption correction: multi-scan (*SADABS*; Bruker, 2008 ▶) *T*_min_ = 0.919, *T*_max_ = 0.936
16250 measured reflections
6594 independent reflections
4827 reflections with *I* > 2σ(*I*)
*R*_int_ = 0.053

#### Refinement

*R*[*F* ^2^ > 2σ(*F* ^2^)] = 0.060
*wR*(*F* ^2^) = 0.123
*S* = 1.00
6594 reflections
476 parameters
15 restraints
H-atom parameters constrained
Δρ_max_ = 0.30 e Å^−3^
Δρ_min_ = −0.28 e Å^−3^
Absolute structure: Flack (1983 ▶)
Flack parameter: 0.04 (7)

### 

Data collection: *SMART* (Bruker, 2008 ▶); cell refinement: *SAINT* (Bruker, 2008 ▶); data reduction: *SAINT*; program(s) used to solve structure: *SHELXS97* (Sheldrick, 2008 ▶); program(s) used to refine structure: *SHELXL97* (Sheldrick, 2008 ▶); molecular graphics: *SHELXTL* (Sheldrick, 2008 ▶); software used to prepare material for publication: *SHELXTL*.

## Supplementary Material

Crystal structure: contains datablock(s) I, New_Global_Publ_Block. DOI: 10.1107/S1600536813018795/ld2101sup1.cif

Structure factors: contains datablock(s) I. DOI: 10.1107/S1600536813018795/ld2101Isup2.hkl

Additional supplementary materials:  crystallographic information; 3D view; checkCIF report

## Figures and Tables

**Table 1:** Hydrogen-bond geometry (Å, °)

*D*—H⋯*A*	*D*—H	H⋯*A*	*D*⋯*A*	*D*—H⋯*A*
O1*W*—H1*WA*⋯Cl1^i^	0.94	2.75	3.208 (6)	111
O1*W*—H1*WA*⋯S2^ii^	0.94	2.88	3.819 (6)	174
O1*W*—H1*WB*⋯Cl2	0.86	2.61	3.240 (7)	132
O3*W*—H3*WA*⋯Cl2^iii^	0.85	2.68	3.275 (10)	129
O3*W*—H3*WB*⋯Cl1^i^	0.85	2.60	3.301 (10)	141
C8—H8⋯Cl1	0.93	2.78	3.664 (5)	159
C10—H10⋯Cl1^iv^	0.93	2.72	3.390 (5)	130
C18—H18⋯O3*W* ^v^	0.93	2.52	3.320 (9)	144
C27—H27⋯Cl1	0.93	2.72	3.642 (5)	175

Symmetry codes: (i) 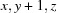; (ii) 
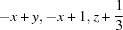
; (iii) 
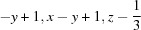
; (iv) 
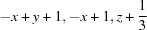
; (v) 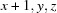.

**Fig. 1.**
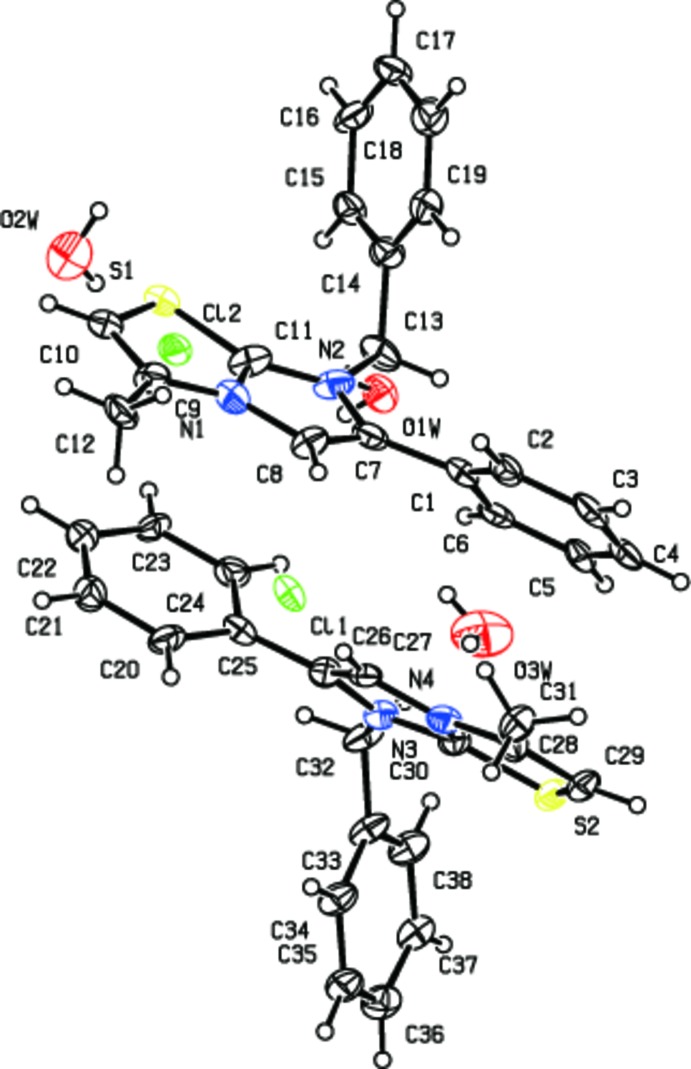
The molecular structure of (I), with atom labels and 30% probability displacement ellipsoids.

**Fig. 2.**
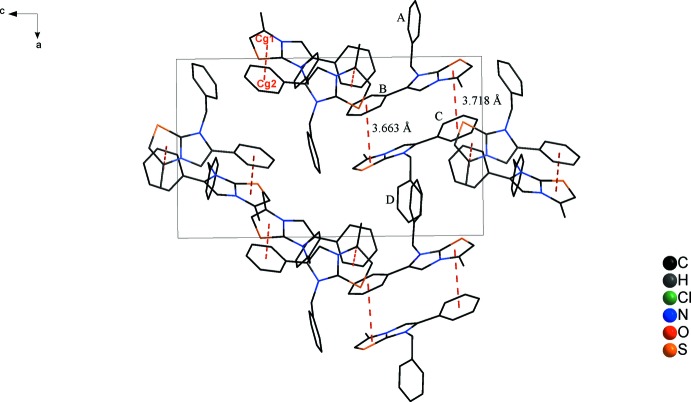
The pi···pi stacking between two symmetrically independent cations.

**Fig. 3.**
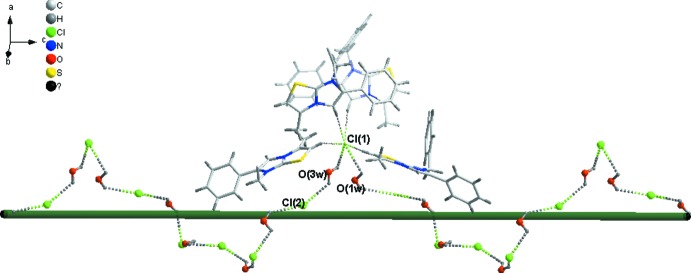
The hydrogen bonded helical anion-water chain and the hydrogen bonding between Cl- ion and the cations.

## supplementary crystallographic information

### Comment

*N*-Heterocyclic carbenes (NHCs) have become ubiquitous ligands in organometallic chemistry and also serve as excellent organocatalysts primarily due to their inherent strong σ-donor ability and nucleophilicity. Recently, Mattson *et al.* (2006), Liu *et al.* (2008), Padmanaban *et al.* (2011) and other researchers have designed new abnormal NHCs compounds and used them as organocatalysts to catalyze umpolung reactions. According to the reports on the synthesis of imidazo[2,1-*b*]thiazoles (Akkurt *et al.* (2011, 2007), Huang *et al.* (2011), Song *et al.* (2008)), herein we report the synthesis and structure of the title compound. The molecular structure of the title compound is depicted in Fig. 1. The crystallographic asymmetric unit of I, contains two 7-benzyl-3-methyl-6-phenyl-imidazo[2,1-*b*]thiazol-7-ium cation, two chlorine anion and three water molecule. As shown in Fig.2, the dihedral angle between benzene ring A and B is 60.85, while C and D is 61.66, indicating the two cations in the unit cell are not equivalent. The two symmetrically independent cations are stabilized by π–π stacking interactions, with a separation of 3.718 and 3.663 Å between the centroids of the benzene and thiazole rings. Another interesting part of the structure of title compound is the helical chain (Cl1—O3w—Cl2—O1w) formed entirely by the O—H···Cl hydrogen-bonding interactions (Fig.3 & Fig.4) hydrogen-bonding interactions along *c* axis in this molecule. O(3w) and O(1w) atoms bridges Cl(1) and Cl(2) atoms with the bond distances of O(3w)···Cl(1) 3.300 (2) Å, O(3w)···Cl(2) 3.275 (1) Å, O(1w)···Cl(1) 3.203 (6) Å, O(1w)···Cl(2) 3.236 (6) Å, while O(2w) doesn't involve in the formation of the helical chain. Probably because O2W water molecules is disordered and isolated, lead to some OH groups without acceptor and can't form hydrogen bonds. The cations connect to the anion-water chain through C—H···Cl hydrogen bonds, and each chloride ion binds to four cations with the average bond distances of 3.389 Å. Thus in the solid-state of title compound, the cation binds to the helical anion-water chain linked by intermolecular hydrogen bonds of O—H···Cl, generating a three-dimensional supramolecular network, and the space between them are occupied by some lattice water molecules.

### Experimental

A mixture of 3-methyl-6-phenylimidazo[2,1-*b*]thiazole (1.071 g, 5.0 mmol) and benzyl chorlide (0.759 g, 6.0 mmol,1.2 equiv) was dissolved in CH3CN, and stirred under reflux for 12 h. The solvent was then removed under vacuum. The white solids obtained were washed with diethyl ether and the crude product was re-crystallized from chloroform / toluene. The yield was 1.45 g, 85%. 1H NMR (δ, 300 MHz, CDCl3): 8.35 (s, 1H), 7.71–7.68 (m, 2H), 7.54–7.52 (m, 3H), 7.41–7.29 (m, 6H), 5.53 (s, 2H), 2.64 (d, J = 1.2 Hz, 3H); 13 C NMR (δ, 75 MHz, CDCl3) 146.9, 140.3, 131.0, 130.8, 130.7, 130.1, 129.8, 129.4, 129.3, 129.1, 125.2, 114.4, 112.3, 51.5, 13.0.

### Refinement

All H atoms attached to carbons were geometrically fixed and allowed to ride on the corresponding non-H atom with C—H = 0.96 Å, and *U*_iso_(H) = 1.5Ueq(C) of the attached C atom for methyl H atoms and 1.2Ueq(C) for other H atoms. Positions of the methyl atoms were optimized rotationally. The water H atoms were located from a Fourier map and their distances were constrained to 0.86 Å and the *U*_iso_(H) = 1.5Ueq(O).

### Crystal data

**Table:**

C_19_H_17_N_2_S^+^·Cl^−^·0.75H_2_O	*D*_x_ = 1.195 Mg m^−^^3^
*M**_r_* = 354.37	Mo *K*α radiation, λ = 0.71073 Å
Trigonal, *P*3_2_	Cell parameters from 25 reflections
Hall symbol: P 32	θ = 1.8–26.0°
*a* = 13.211 (1) Å	µ = 0.31 mm^−^^1^
*c* = 19.555 (3) Å	*T* = 291 K
*V* = 2955.7 (6) Å^3^	Block, colourless
*Z* = 6	0.28 × 0.24 × 0.22 mm
*F*(000) = 1113	

### Data collection

**Table:**

Bruker SMART APEX CCD diffractometer	6594 independent reflections
Radiation source: fine-focus sealed tube	4827 reflections with *I* > 2σ(*I*)
Graphite monochromator	*R*_int_ = 0.053
phi and ω scans	θ_max_ = 26.0°, θ_min_ = 1.8°
Absorption correction: multi-scan (*SADABS*; Bruker, 2008)	*h* = −16→13
*T*_min_ = 0.919, *T*_max_ = 0.936	*k* = −14→16
16250 measured reflections	*l* = −24→16

### Refinement

**Table:**

Refinement on *F*^2^	Hydrogen site location: inferred from neighbouring sites
Least-squares matrix: full	H-atom parameters constrained
*R*[*F*^2^ > 2σ(*F*^2^)] = 0.060	*w* = 1/[σ^2^(*F*_o_^2^) + (0.0606*P*)^2^] where *P* = (*F*_o_^2^ + 2*F*_c_^2^)/3
*wR*(*F*^2^) = 0.123	(Δ/σ)_max_ < 0.001
*S* = 1.00	Δρ_max_ = 0.30 e Å^−^^3^
6594 reflections	Δρ_min_ = −0.28 e Å^−^^3^
476 parameters	Extinction correction: *SHELXL97* (Sheldrick, 2008), Fc^*^=kFc[1+0.001xFc^2^λ^3^/sin(2θ)]^-1/4^
15 restraints	Extinction coefficient: 0.0045 (7)
Primary atom site location: structure-invariant direct methods	Absolute structure: Flack (1983)
Secondary atom site location: difference Fourier map	Flack parameter: 0.04 (7)

### Special details

**Table:**

Geometry. All e.s.d.'s (except the e.s.d. in the dihedral angle between two l.s. planes) are estimated using the full covariance matrix. The cell e.s.d.'s are taken into account individually in the estimation of e.s.d.'s in distances, angles and torsion angles; correlations between e.s.d.'s in cell parameters are only used when they are defined by crystal symmetry. An approximate (isotropic) treatment of cell e.s.d.'s is used for estimating e.s.d.'s involving l.s. planes.
Refinement. Refinement of *F*^2^ against ALL reflections. The weighted *R*-factor *wR* and goodness of fit *S* are based on *F*^2^, conventional *R*-factors *R* are based on *F*, with *F* set to zero for negative *F*^2^. The threshold expression of *F*^2^ > σ(*F*^2^) is used only for calculating *R*-factors(gt) *etc*. and is not relevant to the choice of reflections for refinement. *R*-factors based on *F*^2^ are statistically about twice as large as those based on *F*, and *R*- factors based on ALL data will be even larger.

### Fractional atomic coordinates and isotropic or equivalent isotropic displacement parameters (Å^2^)

**Table:**

	*x*	*y*	*z*	*U*_iso_*/*U*_eq_	Occ. (<1)
C1	0.4467 (3)	0.4753 (4)	0.1467 (2)	0.0415 (10)	
C2	0.4721 (4)	0.4083 (4)	0.1030 (3)	0.0456 (11)	
H2	0.5182	0.3774	0.1174	0.09 (2)*	
C3	0.4258 (4)	0.3880 (4)	0.0358 (3)	0.0523 (12)	
H3	0.4434	0.3455	0.0049	0.068 (17)*	
C4	0.3542 (4)	0.4317 (4)	0.0161 (3)	0.0517 (12)	
H4	0.3223	0.4168	−0.0276	0.035 (11)*	
C5	0.3301 (4)	0.4974 (4)	0.0613 (2)	0.0464 (11)	
H5	0.2827	0.5270	0.0474	0.039 (12)*	
C6	0.3763 (3)	0.5200 (4)	0.1282 (2)	0.0437 (10)	
H6	0.3597	0.5636	0.1588	0.073 (17)*	
C7	0.4878 (4)	0.4879 (4)	0.2189 (2)	0.0454 (11)	
C8	0.4919 (4)	0.4010 (4)	0.2554 (3)	0.0457 (11)	
H8	0.4673	0.3255	0.2403	0.040 (12)*	
C9	0.5679 (4)	0.4122 (4)	0.3779 (3)	0.0441 (10)	
C10	0.6201 (4)	0.5055 (4)	0.4246 (3)	0.0436 (10)	
H10	0.6468	0.4985	0.4674	0.08 (2)*	
C11	0.5674 (4)	0.5640 (4)	0.3202 (3)	0.0527 (12)	
C12	0.5438 (4)	0.2894 (4)	0.3879 (3)	0.0521 (12)	
H12A	0.4609	0.2366	0.3882	0.069 (17)*	
H12B	0.5765	0.2840	0.4307	0.047 (13)*	
H12C	0.5787	0.2690	0.3513	0.12 (3)*	
C13	0.5599 (4)	0.7050 (4)	0.2358 (3)	0.0517 (13)	
H13A	0.5181	0.7308	0.2653	0.051 (14)*	
H13B	0.5305	0.6991	0.1897	0.057 (15)*	
C14	0.6892 (4)	0.7956 (4)	0.2372 (2)	0.0466 (11)	
C15	0.7271 (4)	0.8950 (4)	0.2777 (3)	0.0501 (12)	
H15	0.6742	0.9077	0.3022	0.075 (18)*	
C16	0.8460 (4)	0.9743 (4)	0.2803 (3)	0.0566 (14)	
H16	0.8730	1.0408	0.3073	0.068 (17)*	
C17	0.9239 (4)	0.9570 (4)	0.2442 (3)	0.0577 (13)	
H17	1.0033	1.0116	0.2460	0.077 (18)*	
C18	0.8834 (5)	0.8563 (5)	0.2042 (3)	0.0560 (13)	
H18	0.9361	0.8432	0.1795	0.10 (2)*	
C19	0.7675 (4)	0.7778 (4)	0.2014 (2)	0.0510 (11)	
H19	0.7411	0.7110	0.1747	0.043 (12)*	
C20	0.2128 (4)	0.2665 (4)	0.3611 (2)	0.0458 (10)	
H20	0.1764	0.1878	0.3495	0.072 (17)*	
C21	0.2639 (4)	0.3027 (4)	0.4241 (3)	0.0495 (11)	
H21	0.2614	0.2479	0.4549	0.046 (13)*	
C22	0.3199 (4)	0.4213 (4)	0.4430 (2)	0.0425 (10)	
H22	0.3551	0.4450	0.4857	0.078 (19)*	
C23	0.3219 (3)	0.5016 (4)	0.3973 (2)	0.0393 (9)	
H23	0.3570	0.5800	0.4096	0.072 (17)*	
C24	0.2720 (4)	0.4666 (4)	0.3328 (2)	0.0456 (11)	
H24	0.2763	0.5222	0.3020	0.044 (13)*	
C25	0.2153 (3)	0.3487 (3)	0.3139 (2)	0.0399 (9)	
C26	0.1701 (4)	0.3145 (4)	0.2445 (2)	0.0413 (10)	
C27	0.1880 (3)	0.2417 (4)	0.2030 (2)	0.0403 (10)	
H27	0.2297	0.2044	0.2139	0.056 (15)*	
C28	0.1186 (4)	0.1747 (4)	0.0804 (2)	0.0423 (10)	
C29	0.0565 (4)	0.2059 (4)	0.0319 (2)	0.0423 (10)	
H29	0.0403	0.1797	−0.0130	0.054 (14)*	
C30	0.0853 (3)	0.3017 (4)	0.1444 (2)	0.0378 (7)	
C31	0.1685 (4)	0.0966 (4)	0.0659 (3)	0.0487 (11)	
H31A	0.2388	0.1226	0.0920	0.073*	
H31B	0.1860	0.0996	0.0181	0.073*	
H31C	0.1126	0.0178	0.0785	0.073*	
C32	0.0523 (4)	0.4200 (4)	0.2318 (3)	0.0473 (12)	
H32A	0.0913	0.4940	0.2076	0.09 (2)*	
H32B	0.0730	0.4372	0.2797	0.069 (17)*	
C33	−0.0755 (4)	0.3745 (4)	0.2263 (3)	0.0472 (11)	
C34	−0.1550 (4)	0.2597 (4)	0.2394 (2)	0.0462 (11)	
H34	−0.1282	0.2097	0.2530	0.040 (12)*	
C35	−0.2734 (4)	0.2163 (4)	0.2330 (3)	0.0538 (13)	
H35	−0.3259	0.1375	0.2404	0.064 (16)*	
C36	−0.3133 (4)	0.2921 (4)	0.2153 (3)	0.0510 (12)	
H36	−0.3932	0.2651	0.2137	0.056 (15)*	
C37	−0.2339 (4)	0.4087 (4)	0.1998 (3)	0.0505 (12)	
H37	−0.2602	0.4586	0.1854	0.040 (12)*	
C38	−0.1169 (4)	0.4485 (4)	0.2061 (3)	0.0542 (13)	
H38	−0.0638	0.5265	0.1968	0.060 (15)*	
Cl1	0.34178 (9)	0.08248 (9)	0.23677 (6)	0.0415 (2)	
Cl2	0.50410 (9)	0.85717 (10)	0.41839 (6)	0.0492 (3)	
N1	0.5403 (3)	0.4474 (3)	0.3202 (2)	0.0499 (9)	
N2	0.5361 (3)	0.5880 (3)	0.2585 (2)	0.0486 (10)	
N3	0.1327 (3)	0.2340 (3)	0.14208 (19)	0.0392 (8)	
N4	0.1037 (3)	0.3502 (3)	0.20746 (19)	0.0383 (6)	
O1W	0.4476 (5)	0.9150 (5)	0.2668 (3)	0.0474 (15)	0.50
H1WA	0.4076	0.9367	0.2979	0.057*	0.50
H1WB	0.4754	0.8780	0.2884	0.057*	0.50
O2W	0.7938 (8)	0.2009 (7)	0.4080 (5)	0.072 (2)	0.50
H2WA	0.8587	0.2262	0.3857	0.087*	0.50
H2WB	0.7494	0.2272	0.3924	0.087*	0.50
O3W	0.1192 (8)	0.8296 (9)	0.1880 (6)	0.100 (3)	0.50
H3WA	0.1196	0.7654	0.1890	0.121*	0.50
H3WB	0.1634	0.8744	0.2194	0.121*	0.50
S1	0.63072 (10)	0.63434 (11)	0.39577 (6)	0.0498 (3)	
S2	0.01587 (10)	0.30059 (9)	0.07018 (6)	0.0448 (3)	

### Atomic displacement parameters (Å^2^)

**Table:**

	*U*^11^	*U*^22^	*U*^33^	*U*^12^	*U*^13^	*U*^23^
C1	0.032 (2)	0.037 (2)	0.043 (2)	0.0083 (18)	0.0137 (18)	0.0120 (18)
C2	0.038 (2)	0.044 (2)	0.049 (3)	0.016 (2)	0.0088 (19)	0.022 (2)
C3	0.048 (3)	0.040 (2)	0.049 (3)	0.008 (2)	0.026 (2)	0.015 (2)
C4	0.041 (2)	0.044 (2)	0.046 (3)	0.004 (2)	0.018 (2)	0.016 (2)
C5	0.043 (2)	0.040 (2)	0.043 (3)	0.0101 (19)	0.011 (2)	0.024 (2)
C6	0.0264 (19)	0.035 (2)	0.052 (3)	0.0021 (17)	0.0154 (19)	0.007 (2)
C7	0.031 (2)	0.037 (2)	0.047 (3)	0.0011 (17)	0.0121 (19)	−0.0010 (19)
C8	0.041 (2)	0.042 (2)	0.050 (3)	0.018 (2)	−0.012 (2)	−0.009 (2)
C9	0.035 (2)	0.041 (2)	0.056 (3)	0.0188 (18)	−0.007 (2)	0.012 (2)
C10	0.033 (2)	0.036 (2)	0.048 (3)	0.0079 (18)	0.0031 (19)	0.0041 (19)
C11	0.039 (2)	0.047 (3)	0.069 (3)	0.019 (2)	−0.014 (2)	−0.010 (2)
C12	0.051 (3)	0.036 (2)	0.043 (3)	0.002 (2)	0.016 (2)	0.007 (2)
C13	0.051 (3)	0.028 (2)	0.068 (4)	0.014 (2)	0.016 (2)	0.020 (2)
C14	0.041 (2)	0.035 (2)	0.051 (3)	0.0094 (19)	−0.002 (2)	0.011 (2)
C15	0.042 (2)	0.051 (3)	0.047 (3)	0.015 (2)	0.006 (2)	0.020 (2)
C16	0.048 (3)	0.042 (3)	0.059 (3)	0.008 (2)	−0.028 (2)	−0.002 (2)
C17	0.035 (2)	0.055 (3)	0.063 (3)	0.007 (2)	0.007 (2)	0.023 (3)
C18	0.053 (3)	0.067 (3)	0.051 (3)	0.032 (3)	0.000 (2)	0.002 (2)
C19	0.052 (3)	0.053 (3)	0.037 (3)	0.017 (2)	−0.002 (2)	−0.005 (2)
C20	0.034 (2)	0.043 (2)	0.050 (3)	0.0107 (18)	−0.0118 (19)	−0.0110 (19)
C21	0.054 (3)	0.040 (2)	0.042 (3)	0.015 (2)	−0.001 (2)	0.005 (2)
C22	0.041 (2)	0.040 (2)	0.041 (3)	0.0166 (18)	−0.0011 (19)	0.0033 (18)
C23	0.032 (2)	0.037 (2)	0.041 (2)	0.0120 (18)	−0.0115 (18)	−0.0145 (18)
C24	0.040 (2)	0.033 (2)	0.046 (3)	0.0052 (18)	0.0034 (19)	−0.0038 (19)
C25	0.034 (2)	0.034 (2)	0.044 (2)	0.0115 (17)	0.0133 (18)	0.0089 (17)
C26	0.038 (2)	0.034 (2)	0.040 (2)	0.0092 (18)	−0.0019 (18)	0.0015 (18)
C27	0.0193 (17)	0.048 (2)	0.039 (2)	0.0055 (17)	0.0006 (16)	−0.0057 (19)
C28	0.049 (2)	0.042 (2)	0.033 (2)	0.0211 (19)	0.0004 (19)	0.0074 (18)
C29	0.040 (2)	0.041 (2)	0.038 (2)	0.0144 (18)	−0.0121 (18)	−0.0122 (18)
C30	0.0324 (14)	0.0343 (14)	0.0412 (16)	0.0124 (12)	−0.0032 (13)	0.0015 (12)
C31	0.053 (3)	0.051 (3)	0.044 (3)	0.028 (2)	−0.012 (2)	−0.013 (2)
C32	0.035 (2)	0.055 (3)	0.055 (3)	0.025 (2)	−0.019 (2)	−0.027 (2)
C33	0.043 (2)	0.041 (2)	0.056 (3)	0.020 (2)	−0.024 (2)	−0.018 (2)
C34	0.047 (3)	0.031 (2)	0.056 (3)	0.0168 (19)	−0.017 (2)	−0.011 (2)
C35	0.048 (3)	0.047 (3)	0.048 (3)	0.010 (2)	−0.008 (2)	−0.016 (2)
C36	0.048 (3)	0.043 (2)	0.055 (3)	0.018 (2)	−0.006 (2)	−0.020 (2)
C37	0.047 (3)	0.054 (3)	0.050 (3)	0.025 (2)	−0.014 (2)	−0.011 (2)
C38	0.051 (3)	0.045 (3)	0.058 (3)	0.018 (2)	−0.016 (2)	−0.018 (2)
Cl1	0.0466 (6)	0.0407 (5)	0.0405 (5)	0.0243 (5)	0.0168 (4)	0.0141 (4)
Cl2	0.0390 (5)	0.0467 (6)	0.0495 (7)	0.0121 (5)	−0.0010 (5)	0.0196 (5)
N1	0.046 (2)	0.046 (2)	0.048 (2)	0.0151 (17)	0.0070 (17)	0.0076 (17)
N2	0.0363 (19)	0.0349 (19)	0.066 (3)	0.0113 (16)	−0.0117 (18)	−0.0056 (18)
N3	0.0315 (17)	0.0290 (16)	0.048 (2)	0.0081 (14)	0.0022 (14)	−0.0012 (15)
N4	0.0330 (13)	0.0344 (14)	0.0434 (15)	0.0137 (11)	−0.0028 (12)	−0.0029 (11)
O1W	0.055 (4)	0.043 (3)	0.046 (4)	0.026 (3)	0.011 (3)	0.001 (3)
O2W	0.092 (6)	0.058 (4)	0.078 (6)	0.045 (4)	−0.012 (5)	−0.007 (4)
O3W	0.069 (5)	0.114 (7)	0.138 (9)	0.060 (6)	0.011 (5)	−0.027 (7)
S1	0.0370 (6)	0.0464 (6)	0.0500 (7)	0.0089 (5)	0.0067 (5)	0.0070 (5)
S2	0.0416 (6)	0.0377 (5)	0.0446 (6)	0.0119 (5)	−0.0134 (5)	−0.0021 (5)

### Geometric parameters (Å, º)

**Table:**

C1—C6	1.376 (7)	C21—C22	1.406 (6)
C1—C2	1.387 (7)	C21—H21	0.9300
C1—C7	1.493 (6)	C22—C23	1.378 (6)
C2—C3	1.417 (7)	C22—H22	0.9300
C2—H2	0.9300	C23—C24	1.389 (6)
C3—C4	1.385 (7)	C23—H23	0.9300
C3—H3	0.9300	C24—C25	1.399 (6)
C4—C5	1.383 (7)	C24—H24	0.9300
C4—H4	0.9300	C25—C26	1.460 (6)
C5—C6	1.411 (7)	C26—C27	1.365 (6)
C5—H5	0.9300	C26—N4	1.390 (6)
C6—H6	0.9300	C27—N3	1.374 (6)
C7—C8	1.376 (7)	C27—H27	0.9300
C7—N2	1.382 (6)	C28—N3	1.399 (6)
C8—N1	1.413 (6)	C28—C29	1.441 (6)
C8—H8	0.9300	C28—C31	1.504 (7)
C9—N1	1.339 (6)	C29—S2	1.756 (5)
C9—C10	1.407 (7)	C29—H29	0.9300
C9—C12	1.502 (6)	C30—N3	1.324 (5)
C10—S1	1.731 (5)	C30—N4	1.354 (6)
C10—H10	0.9300	C30—S2	1.713 (4)
C11—N2	1.363 (7)	C31—H31A	0.9600
C11—N1	1.395 (6)	C31—H31B	0.9600
C11—S1	1.723 (5)	C31—H31C	0.9600
C12—H12A	0.9600	C32—N4	1.471 (5)
C12—H12B	0.9600	C32—C33	1.486 (6)
C12—H12C	0.9600	C32—H32A	0.9700
C13—N2	1.483 (6)	C32—H32B	0.9700
C13—C14	1.519 (6)	C33—C34	1.370 (6)
C13—H13A	0.9700	C33—C38	1.393 (7)
C13—H13B	0.9700	C34—C35	1.377 (7)
C14—C19	1.362 (7)	C34—H34	0.9300
C14—C15	1.396 (7)	C35—C36	1.388 (8)
C15—C16	1.386 (7)	C35—H35	0.9300
C15—H15	0.9300	C36—C37	1.396 (7)
C16—C17	1.358 (8)	C36—H36	0.9300
C16—H16	0.9300	C37—C38	1.367 (7)
C17—C18	1.399 (8)	C37—H37	0.9300
C17—H17	0.9300	C38—H38	0.9300
C18—C19	1.355 (7)	O1W—H1WA	0.9407
C18—H18	0.9300	O1W—H1WB	0.8555
C19—H19	0.9300	O2W—H2WA	0.8657
C20—C21	1.370 (6)	O2W—H2WB	0.8731
C20—C25	1.414 (6)	O3W—H3WA	0.8501
C20—H20	0.9300	O3W—H3WB	0.8502
			
C6—C1—C2	122.9 (4)	C22—C23—C24	120.6 (4)
C6—C1—C7	119.1 (4)	C22—C23—H23	119.7
C2—C1—C7	117.7 (4)	C24—C23—H23	119.7
C1—C2—C3	118.2 (4)	C23—C24—C25	120.8 (4)
C1—C2—H2	120.9	C23—C24—H24	119.6
C3—C2—H2	120.9	C25—C24—H24	119.6
C4—C3—C2	119.9 (5)	C24—C25—C20	118.4 (4)
C4—C3—H3	120.1	C24—C25—C26	120.0 (4)
C2—C3—H3	120.1	C20—C25—C26	121.3 (4)
C5—C4—C3	120.3 (5)	C27—C26—N4	106.9 (4)
C5—C4—H4	119.9	C27—C26—C25	125.7 (4)
C3—C4—H4	119.9	N4—C26—C25	127.5 (4)
C4—C5—C6	120.9 (5)	C26—C27—N3	107.2 (4)
C4—C5—H5	119.5	C26—C27—H27	126.4
C6—C5—H5	119.5	N3—C27—H27	126.4
C1—C6—C5	117.8 (5)	N3—C28—C29	110.0 (4)
C1—C6—H6	121.1	N3—C28—C31	124.4 (4)
C5—C6—H6	121.1	C29—C28—C31	125.5 (4)
C8—C7—N2	108.4 (4)	C28—C29—S2	110.1 (3)
C8—C7—C1	124.2 (4)	C28—C29—H29	124.9
N2—C7—C1	127.3 (4)	S2—C29—H29	124.9
C7—C8—N1	107.5 (4)	N3—C30—N4	108.5 (4)
C7—C8—H8	126.2	N3—C30—S2	113.4 (3)
N1—C8—H8	126.2	N4—C30—S2	138.1 (3)
N1—C9—C10	110.0 (4)	C28—C31—H31A	109.5
N1—C9—C12	122.6 (4)	C28—C31—H31B	109.5
C10—C9—C12	127.4 (4)	H31A—C31—H31B	109.5
C9—C10—S1	114.4 (4)	C28—C31—H31C	109.5
C9—C10—H10	122.8	H31A—C31—H31C	109.5
S1—C10—H10	122.8	H31B—C31—H31C	109.5
N2—C11—N1	108.4 (4)	N4—C32—C33	120.7 (4)
N2—C11—S1	139.0 (4)	N4—C32—H32A	107.2
N1—C11—S1	112.5 (4)	C33—C32—H32A	107.2
C9—C12—H12A	109.5	N4—C32—H32B	107.2
C9—C12—H12B	109.5	C33—C32—H32B	107.2
H12A—C12—H12B	109.5	H32A—C32—H32B	106.8
C9—C12—H12C	109.5	C34—C33—C38	118.5 (4)
H12A—C12—H12C	109.5	C34—C33—C32	121.4 (4)
H12B—C12—H12C	109.5	C38—C33—C32	120.1 (4)
N2—C13—C14	112.6 (4)	C33—C34—C35	121.7 (5)
N2—C13—H13A	109.1	C33—C34—H34	119.2
C14—C13—H13A	109.1	C35—C34—H34	119.2
N2—C13—H13B	109.1	C34—C35—C36	119.0 (5)
C14—C13—H13B	109.1	C34—C35—H35	120.5
H13A—C13—H13B	107.8	C36—C35—H35	120.5
C19—C14—C15	120.5 (4)	C35—C36—C37	120.2 (5)
C19—C14—C13	120.5 (4)	C35—C36—H36	119.9
C15—C14—C13	118.9 (4)	C37—C36—H36	119.9
C16—C15—C14	118.0 (5)	C38—C37—C36	119.1 (5)
C16—C15—H15	121.0	C38—C37—H37	120.5
C14—C15—H15	121.0	C36—C37—H37	120.5
C17—C16—C15	121.4 (5)	C37—C38—C33	121.4 (5)
C17—C16—H16	119.3	C37—C38—H38	119.3
C15—C16—H16	119.3	C33—C38—H38	119.3
C16—C17—C18	119.3 (5)	C9—N1—C11	114.8 (4)
C16—C17—H17	120.3	C9—N1—C8	138.3 (4)
C18—C17—H17	120.3	C11—N1—C8	106.9 (4)
C19—C18—C17	119.9 (5)	C11—N2—C7	108.8 (4)
C19—C18—H18	120.0	C11—N2—C13	125.2 (4)
C17—C18—H18	120.0	C7—N2—C13	125.8 (4)
C18—C19—C14	120.8 (5)	C30—N3—C27	109.5 (4)
C18—C19—H19	119.6	C30—N3—C28	115.6 (4)
C14—C19—H19	119.6	C27—N3—C28	134.9 (4)
C21—C20—C25	120.1 (4)	C30—N4—C26	107.9 (3)
C21—C20—H20	120.0	C30—N4—C32	124.2 (4)
C25—C20—H20	120.0	C26—N4—C32	127.7 (4)
C20—C21—C22	121.2 (4)	H1WA—O1W—H1WB	108.8
C20—C21—H21	119.4	H2WA—O2W—H2WB	113.8
C22—C21—H21	119.4	H3WA—O3W—H3WB	109.5
C23—C22—C21	118.9 (4)	C11—S1—C10	88.3 (2)
C23—C22—H22	120.5	C30—S2—C29	90.8 (2)
C21—C22—H22	120.5		
			
C6—C1—C2—C3	1.9 (6)	C36—C37—C38—C33	1.3 (8)
C7—C1—C2—C3	175.1 (3)	C34—C33—C38—C37	0.4 (8)
C1—C2—C3—C4	−2.1 (6)	C32—C33—C38—C37	179.2 (5)
C2—C3—C4—C5	1.6 (6)	C10—C9—N1—C11	−0.8 (6)
C3—C4—C5—C6	−0.8 (6)	C12—C9—N1—C11	179.1 (4)
C2—C1—C6—C5	−1.1 (6)	C10—C9—N1—C8	177.4 (5)
C7—C1—C6—C5	−174.2 (3)	C12—C9—N1—C8	−2.7 (9)
C4—C5—C6—C1	0.5 (6)	N2—C11—N1—C9	179.3 (4)
C6—C1—C7—C8	138.1 (5)	S1—C11—N1—C9	0.0 (5)
C2—C1—C7—C8	−35.4 (6)	N2—C11—N1—C8	0.6 (5)
C6—C1—C7—N2	−47.5 (6)	S1—C11—N1—C8	−178.7 (3)
C2—C1—C7—N2	138.9 (5)	C7—C8—N1—C9	−179.9 (5)
N2—C7—C8—N1	2.0 (5)	C7—C8—N1—C11	−1.6 (5)
C1—C7—C8—N1	177.3 (4)	N1—C11—N2—C7	0.7 (5)
N1—C9—C10—S1	1.3 (5)	S1—C11—N2—C7	179.6 (5)
C12—C9—C10—S1	−178.6 (4)	N1—C11—N2—C13	−174.3 (4)
N2—C13—C14—C19	56.6 (6)	S1—C11—N2—C13	4.6 (9)
N2—C13—C14—C15	−120.9 (5)	C8—C7—N2—C11	−1.7 (5)
C19—C14—C15—C16	0.0 (7)	C1—C7—N2—C11	−176.8 (4)
C13—C14—C15—C16	177.5 (4)	C8—C7—N2—C13	173.3 (4)
C14—C15—C16—C17	0.6 (7)	C1—C7—N2—C13	−1.8 (7)
C15—C16—C17—C18	−0.9 (8)	C14—C13—N2—C11	57.4 (6)
C16—C17—C18—C19	0.6 (8)	C14—C13—N2—C7	−116.8 (5)
C17—C18—C19—C14	0.0 (8)	N4—C30—N3—C27	−2.9 (4)
C15—C14—C19—C18	−0.3 (8)	S2—C30—N3—C27	179.0 (3)
C13—C14—C19—C18	−177.7 (5)	N4—C30—N3—C28	178.4 (3)
C25—C20—C21—C22	−0.3 (7)	S2—C30—N3—C28	0.3 (5)
C20—C21—C22—C23	0.7 (7)	C26—C27—N3—C30	1.9 (4)
C21—C22—C23—C24	−1.6 (7)	C26—C27—N3—C28	−179.8 (4)
C22—C23—C24—C25	2.1 (7)	C29—C28—N3—C30	1.7 (5)
C23—C24—C25—C20	−1.7 (6)	C31—C28—N3—C30	177.9 (4)
C23—C24—C25—C26	−176.0 (4)	C29—C28—N3—C27	−176.5 (4)
C21—C20—C25—C24	0.8 (7)	C31—C28—N3—C27	−0.3 (7)
C21—C20—C25—C26	175.0 (4)	N3—C30—N4—C26	2.8 (4)
C24—C25—C26—C27	131.4 (4)	S2—C30—N4—C26	−179.8 (4)
C20—C25—C26—C27	−42.8 (6)	N3—C30—N4—C32	−172.8 (4)
C24—C25—C26—N4	−47.3 (6)	S2—C30—N4—C32	4.6 (7)
C20—C25—C26—N4	138.6 (4)	C27—C26—N4—C30	−1.6 (4)
N4—C26—C27—N3	−0.1 (4)	C25—C26—N4—C30	177.2 (4)
C25—C26—C27—N3	−179.0 (4)	C27—C26—N4—C32	173.7 (4)
N3—C28—C29—S2	−2.9 (4)	C25—C26—N4—C32	−7.4 (7)
C31—C28—C29—S2	−179.1 (4)	C33—C32—N4—C30	51.6 (7)
N4—C32—C33—C34	40.2 (7)	C33—C32—N4—C26	−123.1 (5)
N4—C32—C33—C38	−138.5 (5)	N2—C11—S1—C10	−178.3 (6)
C38—C33—C34—C35	0.2 (7)	N1—C11—S1—C10	0.6 (4)
C32—C33—C34—C35	−178.6 (5)	C9—C10—S1—C11	−1.0 (4)
C33—C34—C35—C36	−2.5 (7)	N3—C30—S2—C29	−1.8 (3)
C34—C35—C36—C37	4.2 (7)	N4—C30—S2—C29	−179.1 (5)
C35—C36—C37—C38	−3.7 (7)	C28—C29—S2—C30	2.7 (3)

### Hydrogen-bond geometry (Å, º)

**Table:**

*D*—H···*A*	*D*—H	H···*A*	*D*···*A*	*D*—H···*A*
O1*W*—H1*WA*···Cl1^i^	0.94	2.75	3.208 (6)	111
O1*W*—H1*WA*···S2^ii^	0.94	2.88	3.819 (6)	174
O1*W*—H1*WB*···Cl2	0.86	2.61	3.240 (7)	132
O3*W*—H3*WA*···Cl2^iii^	0.85	2.68	3.275 (10)	129
O3*W*—H3*WB*···Cl1^i^	0.85	2.60	3.301 (10)	141
C8—H8···Cl1	0.93	2.78	3.664 (5)	159
C10—H10···Cl1^iv^	0.93	2.72	3.390 (5)	130
C18—H18···O3*W*^v^	0.93	2.52	3.320 (9)	144
C27—H27···Cl1	0.93	2.72	3.642 (5)	175

Symmetry codes: (i) *x*, *y*+1, *z*; (ii) −*x*+*y*, −*x*+1, *z*+1/3; (iii) −*y*+1, *x*−*y*+1, *z*−1/3; (iv) −*x*+*y*+1, −*x*+1, *z*+1/3; (v) *x*+1, *y*, *z*.
